# Pharmacist involvement in the inhaler choice improves lung function in patients with COPD: a prospective single-arm study

**DOI:** 10.1186/s40780-021-00211-0

**Published:** 2021-08-02

**Authors:** Eiji Shiwaku, Satoshi Dote, Shinobu Kaneko, Chisato Hei, Masaki Aikawa, Yuki Sakai, Takahiro Kawai, Shigeaki Iwatsubo, Michinobu Hashimoto, Teppei Tsuneishi, Tomoko Nishimura, Toshiyuki Iwata, Daiki Hira, Tomohiro Terada, Takashi Nishimura, Yuka Kobayashi

**Affiliations:** 1 Department of Pharmacy, Kyoto-Katsura Hospital, 17, Yamadahiraocho, Kyoto-shi Nishikyo-ku, Kyoto, 615-8256 Japan; 2 Department of Respiratory Medicine, Kyoto-Katsura Hospital, Kyoto, Japan; 3 Department of Pharmacy, Shiga University of Medical Science Hospital, Shiga, Japan; 4 College of Pharmaceutical Sciences, Ritsumeikan University, Shiga, Japan

**Keywords:** Chronic obstructive pulmonary disease, Inhalation devices, Pharmaceutical care, Medication therapy management, Shared decision-making

## Abstract

**Background:**

Currently, in Japan, shifting tasks from physician to hospital pharmacist is being developed to reduce physician workload and improve the quality of pharmacotherapy. This study aimed to investigate the effects of pharmacist involvement in the choice of inhaler as the task on the clinical outcomes of patients with chronic obstructive pulmonary disease (COPD).

**Methods:**

This prospective, single-center, single-arm study included 36 outpatients with newly diagnosed COPD indicating inhaler therapy. Eligible patients were immediately interviewed by pharmacist. Then, pharmacist assessed patient’s inhalation flow rate, physical function to handle an inhaler, comprehension, and value, and finally recommended a personalized inhaler based on originally developed inhaler choice protocol, and pulmonologist prescribed a pharmacist-selected inhaler. The primary endpoint was the improvement in trough forced expiratory volume in 1 s (FEV_1_) between baseline and week 26. The secondary endpoints were safety, and improvements at week 26 in scores for the COPD Assessment Test (CAT), modified British Medical Research Council Dyspnea Scale (mMRC), and Adherence Starts with Knowledge-20 (ASK-20).

**Results:**

The pneumonologists completely agreed with the pharmacist-recommended inhaler. Mean FEV_1_ significantly increased from baseline to week 26 (1.60, SD 0.54 L vs. 1.98, SD 0.56 L; *p* < 0.0001). Significant improvements in CAT, mMRC, and ASK-20 scores were also observed. The prevalence of CAT responders as a negative predictor of acute exacerbation, defined as those with a decrease in CAT score of ≥2 points from baseline, was 86%. None of the patients experienced exacerbation during the study period.

**Conclusions:**

Pharmacist involvement in the choice of inhaler for patients with newly diagnosed COPD was associated with improved lung function, health status, clinical symptoms, and adherence to inhaler therapy. Shifting task of choosing appropriate inhaler from physician to hospital pharmacist may be performed effectively and safely with an inhaler choice protocol.

**Trial registration number:**

UMIN000039722, retrospectively registered on March 10, 2020.

**Supplementary Information:**

The online version contains supplementary material available at 10.1186/s40780-021-00211-0.

## Introduction

Pharmacological therapy, whether by inhaler or oral medication, has an important role in the management of chronic obstructive pulmonary disease (COPD), helping to reduce symptoms, prevent exacerbation and improve exercise tolerance and health status. Inhaler therapies include bronchodilators, such as β_2_-agonists and anticholinergics as well as corticosteroids. Unlike oral medications, the use of inhalers for COPD is associated with some problems. First, patients with COPD need to continue their inhaled medication over a long period, and non-adherence is a common problem [1–3)]. Second, most patients are elderly in Japan, presenting challenges with handling inhalers [4, 5)]. Reported risk factors for non-adherence to inhaled medication include a low Global Initiative for Chronic Obstructive Lung Disease (GOLD) stage [6)] and patient-related factors such as knowledge, attitudes, beliefs, perceptions and expectations [7)]. To address this, studies have highlighted the importance of patient education, such as proper use of inhalers and purpose of medication [8)], and shared decision-making [9)]. However, these studies did not examine the impact of patient education and shared decision-making on the improvement of lung function in COPD patients.

In Japan, pharmacists cannot prescribe medication and, conventionally, play a role in enhancing patients’ medication adherence after appropriate medicines are prescribed. However, there is now an initiative, known as protocol-based pharmacotherapy management (PBPM), in which pharmacists manage medication in collaboration with physicians; this is similar to collaborative drug therapy management in the USA [10)]. PBPM has already shown some success; for example, Katada et al. reported improvement in the percentage of time patients were within the therapeutic range during the first 10 days of warfarin therapy [11)]. In the respiratory field, Hokoyama et al. reported that the collaborative team, which consisted of pulmonologist, hospital- and community-pharmacists, services reduced the hospitalization due to acute exacerbation. The authors also mentioned future perspective about the shifting task of choosing inhaler from physician to hospital pharmacist under the PBPM concept [12)].

Applying the PBPM concept to COPD inhaler therapy, we hypothesized that pharmacist-managed personalized inhaler therapy, in which the pharmacist leads the choice of the most suitable inhaler for the patient, may result in improved outcomes as well as medication adherence. The aim of this study, therefore, was to investigate the feasibility of shifting task of choosing inhaler from physician to hospital pharmacist with the outcomes of COPD patients.

## Methods

This single-center, prospective, single-arm study enrolled outpatients with newly diagnosed COPD that required newly planned inhaler therapy, who attended the Department of Respiratory Medicine, Kyoto-Katsura Hospital between 1 April 2016 and 31 March 2018. Eligible patients were those with stable COPD, defined as the presence of a post-bronchodilator forced expiratory volume in 1 s (FEV_1_) of < 70% and confirmation from the physician that they were clinically stable. The study excluded any patients who had undergone pneumonectomy or who had dementia, lung cancer, or interstitial pneumonia and could not be provided with caregiving support for inhaler therapy.

**Fig. 1:**
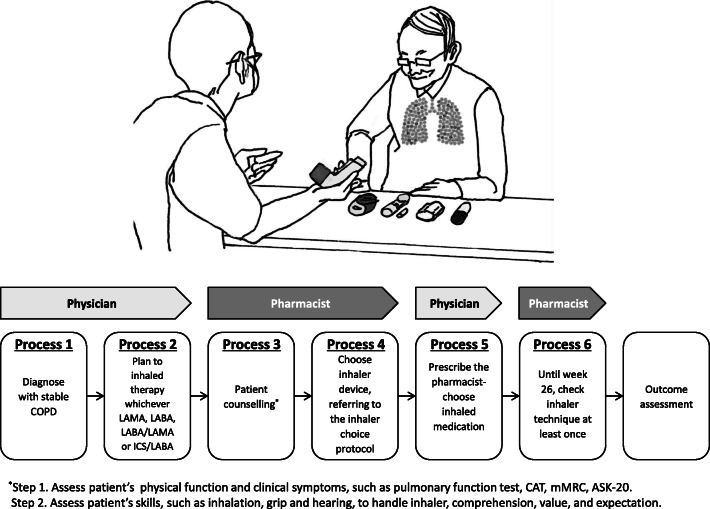
The study design. Abbreviations: COPD, chronic obstructive pulmonary disease; CAT, COPD Assessment Test, mMRC, modified British Medical Research Council Dyspnea Scale; ASK-20, Adherence Starts with Knowledge-20, LAMA, long-acting muscarinic antagonist; LABA, long-acting beta 2-agonist; ICS, inhaled corticosteroid

**Fig. 2:**
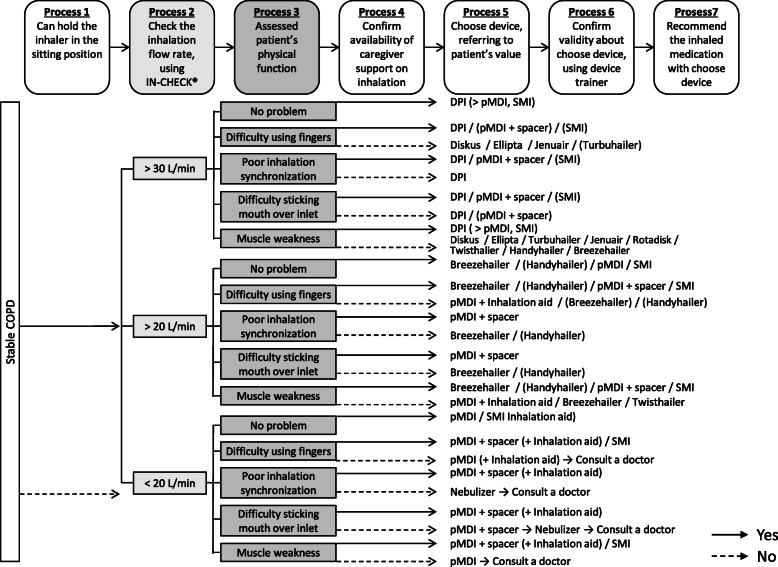
The inhaler choice protocol**.** Solid arrows indicate ‘Yes’ and dotted arrows indicate ‘No’. IN-CHECK^™^ (Clement Clarke International Ltd., Harlow, England) was used to evaluate the peak inspiratory flow in Process 2. The pharmacists assessed patient’s physical function subjectively in Process 3. ‘DPI (> pMDI, SMI)’ indicates that DPI should be prioritized over pMDI or SMI unless there is a specific reason otherwise. Aero Chamber^®^ Plus Flow-Vu^®^ (Allergan, Inc., Dublin, Ireland) was used as a spacer device Abbreviations: COPD, chronic obstructive pulmonary disease; DPI, dry powder inhaler; pMDI, pressurized metered-dose inhaler; SMI, soft mist inhaler.

The study design is shown in Fig. 1. After receiving a diagnosis of COPD that indicated inhaler therapy, the patient was immediately interviewed by the hospital pharmacist (ES, CH, or SK), who assessed the patient’s ability to use an inhaler [considering factors such as inhalation, grip and hearing (e.g., a whirring sound should be heard for Breezehaler^®^)] and his or her understanding of what is required, as well as the patient’s expectations and preferences, and practical considerations such as device portability, the need for visual and auditory confirmation of successful inhalation, daily dose frequency and medication cost. Thus, the patient could choice his or her inhaler with the pharmacist. Based on this, the pharmacist selected the most suitable inhaler by referring to the inhaler choice protocol originally developed via the discussion between the pharmacists (ES, CH, and SK) and the pulmonologist (MA, YS, TK, SI, MH, TT, TN, TI and TN) focusing the continuity of inhaler therapy (Fig. 2). When the proposal is reasonable, the physician finally prescribed the pharmacist-selected inhaler.

The study period for each patient was 26 weeks (±2 weeks) from the commencement of inhaler use (baseline) referring to the previous study [13)]. Prior to this, the patient’s FEV_1_ was measured and his or her status was assessed using three scales. The COPD Assessment Test (CAT) [14)] is a quick and easy self-completed test for COPD patients, which provides a score indicating the impact of the disease on their health status. Possible scores are in the range 0–40, with high scores representing poor health status. The modified British Medical Research Council Dyspnea Scale (mMRC) [15)] is widely used for the assessment of dyspnea in COPD patients. Possible scores are 0–5, with high scores representing worse dyspnea. Adherence Starts with Knowledge-20 (ASK-20) [16, 17)] is a 20-item survey that identifies actionable risk factors for non-adherence to medication [18)]. At the end of the study period, the patient’s trough FEV_1_ was measured and the three scales were re-administered.

The primary endpoint was the change in FEV_1_ between baseline and the end of the study period. The secondary endpoints were safety and the changes in the scores for the three scales between baseline and the end of the study period. Regarding the ASK-20, ‘with barrier’ frequency, previously defined by Steven et al. [17)], were also analysed.

This study was performed in accordance with the Declaration of Helsinki and its amendments and was approved by the Ethics Committee of Kyoto-Katsura Hospital (Approval number: 448). All the participants gave their written informed consent.

### Statistical analysis

A sample size estimate indicated that a sample of 60 patients would be sufficient to achieve 80% power to detect a significant (*p* < 0.05) change in the primary endpoint between baseline and the end of the study period. This assumed a change in FEV_1_ of 0.3 L with a standard deviation of 0.15 L, based on a clinically significant change of 0.1 L [19)] plus an expected effect size for the inhaled medication of 0.2 L. [20)] The estimated dropout rate was 25%. After completion of the study, the exclusion criteria were amended to improve the internal validity of study by limiting to the homogeneous participants, i.e., outpatient and patient without lung cancer or interstitial pneumonia. Finally, 36 patients were analysed. A safety analysis was performed for all the enrolled patients because of uncertainty about the safety of the shifting task from physician to hospital pharmacist.

Statistical analyses were performed using GraphPad Prism for Windows version 8.2.1 (GraphPad Software, San Diego, CA, USA). Binary outcomes were compared using Fisher’s exact test, and continuous outcomes were compared using paired t-test, or Mann–Whitney U test, as appropriate.

## Results

### Patient characteristics

The patient flow diagram is shown in Supplementary Fig. S1. A total of 60 patients were initially enrolled, of which 36 (60%) were analysed. Patient characteristics are shown in Table 1. The reasons for the pharmacist’s choice of inhaler for each patient are shown in Supplementary Table S1. The pulmonologists completely agreed with the pharmacists-recommended inhaler. The prevalence of each type of prescribed inhaler are shown in Supplementary Table S2. Regarding the outpatient visit frequency until study end (patients invariably visit twice, i.e., baseline and week 26), mean number of visits was 3.5 (SD, 0.6) times. In other words, the additional visit to check inhaler technique (Fig. 1, Process 6) was 1.5 times. The median time taken by the pharmacist to complete the counselling of each patient in the choice of inhaler was 30 (range, 20―54) min.

**Table 1:** Patient characteristics at baseline

Variable	*n* = 36
Age, years	71.7 ± 9.1
Male sex, n (%)	31 (86.1)
ECOG performance status, n (%)	
0	29 (80.6)
1	4 (11.1)
2	1 (2.8)
3	2 (5.6)
Body weight, kg	62.0 ± 10.9
Body surface area, m^2^	1.68 ± 0.17
FEV_1_, L	1.60 ± 0.54
Percentage of predicted FEV_1_ (%)	59.69 ± 16.35
COPD stage, n (%)^*^	
Stage I	4 (11.1)
Stage II	21 (58.3)
Stage III	10 (27.8)
Stage IV	1 (2.8)
GOLD 2017 category, n (%)	
Group A	6 (16.7)
Group B	19 (52.8)
Group C	1 (2.8)
Group D	10 (27.7)
Smoking status, n (%)	
Smoking history, pack-years	53.5 ± 27.36
Never / former / current smoker	0 / 32 / 4
Prior history of exacerbation within 1 year, n (%)	
0 exacerbations	36 (100)
1 exacerbation	0

Abbreviations: *ECOG* Eastern Cooperative Oncology Group performance status, *FEV*_*1*_ forced expiratory volume in 1 s. Data are presented as mean ± SD. ^*^COPD stage (based on post-bronchodilator FEV_1_) is as follows: Stage I indicates FEV_1_ ≥ 80% predicted; Stage II, 50% ≤ FEV_1_ < 80% predicted; Stage III, 30% ≤ FEV_1_ < 50% predicted; and Stage IV, FEV_1_ < 30% predicted

### Primary endpoint: changes in FEV_1_

**Fig. 3:**
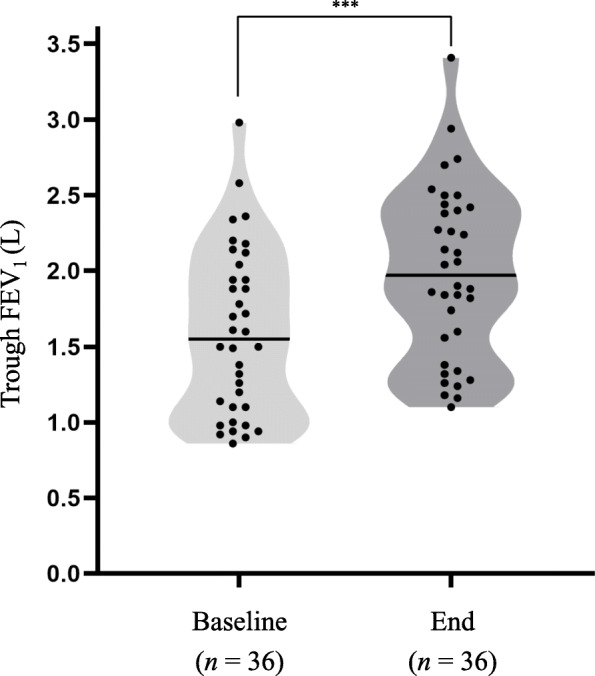
Violin plot depicting the distribution of FEV_1_ values at baseline and the end of the study period (week 26 ± 2). The shape of the violin plot shows the increased frequency of the corresponding FEV_1_ distribution. The dots indicate the individual patients, and the bars indicate the median values. Mean FEV_1_ significantly improved from baseline to week 26 (1.60, SD 0.54 L vs. 1.98, SD 0.56 L; p < 0.0001). Abbreviation: FEV_1_ forced expiratory volume in 1 s

The mean improvement in FEV_1_ from baseline to week 26 was 0.39, SD 0.29 L (95% confidence interval: 0.26 to 0.49; *p* < 0.0001) (Fig. 3). A subgroup analysis of the change in FEV_1_ according to the patients’ COPD stage showed consistent improvement in all COPD stages (Supplementary Table S3).

### Secondary endpoints: safety and changes in CAT, mMRC and ASK-20 scores

Adverse events were experienced by eight (13%) of the initial 60 patients in the pharmacist involvement in the inhaler choice group, as follows: dry mouth (4 patients, 7%), hoarseness (3 patients, 5%) and ischuria (one patient, 2%). During the median 26 weeks (range 12–28 weeks) of follow-up, no patient experienced acute exacerbation.

Table 2 shows the changes from baseline to the end of the study period in the CAT and mMRC scores. CAT and mMRC scores both improved significantly. In a post hoc analysis, the prevalence of CAT responders, defined as those with a decrease of ≥2 points from baseline, was 86% (31/36). The percentage of patients with mMRC dyspnea scores ≥2 points decreased significantly from 72% at baseline to 17% at the end of the study period (*P* < 0.001). Table 3 summaries the changes in ASK-20 scores. There were significant improvements in the domains Attitudes and Beliefs, Help from Others, Talking with Healthcare Team, and Taking Medicines. Especially, the barriers with reaching health goals and shared decision-making were largely removed (P < 0.001).

**Table 2:** Changes in CAT and mMRC scores from baseline to the end of the study period

	Baseline (*n* = 36)	End (*n* = 36)	*P* value
CAT	12.31 ± 7.79	4.39 ± 3.20	< 0.0001
mMRC	2.53 ± 1.48	1.17 ± 0.83	< 0.0001

Data are presented as mean ± SD. The study period was 26 ± 2 weeks. Abbreviations: *CAT* chronic obstructive pulmonary disease assessment test, *mMRC* Modified Medical Research Council dyspnea scale

**Table 3:** Changes in ASK-20 scores from baseline to the end of the study period

ASK-20 components	Baseline (*n* = 36)			End (*n* = 36)			P^*^	*P*^‡^
	Score		Barriers	Score		Barriers		
	Mean	SD	n (%)	Mean	SD	n (%)		
**Lifestyle**								
Q1 I just forget to take my medicines some of the time.	1.83	1.26	7 (19.4)	1.61	1.03	4 (11.1)	0.42	1.0
Q2 I run out of my medicine because I don’t get refills on time.	1.28	0.84	2 (5.6)	1.06	0.23	0	0.13	0.53
Q3 My use of alcohol gets in the way of taking my medicines.	1.11	0.39	0	1.06	0.23	0	0.47	1.0
Q4 I worry about how medicine will affect my sexual health.	1.03	0.16	0	1.03	0.16	0	1.0	1.0
Q5 I sometimes forget things that are important to me.	2.25	1.34	8 (22.2)	1.67	0.94	3 (8.3)	0.04	< 0.001
Q6 I have felt sad, down, or blue during the past month.	1.92	1.30	3 (8.3)	1.36	0.85	0	0.04	0.29
**Attitudes and Beliefs**								
Q7 I feel confident that each one of my medicines will help me.	2.17	1.32	11 (30.6)	1.36	0.58	1(2.8)	< 0.01	< 0.01
Q8 I know if I am reaching my health goals.	3.08	1.21	24 (66.7)	1.72	0.65	4 (11.1)	< 0.0001	< 0.001
**Help from Others**								
Q9 I have someone I can call with questions about my medicines.	2.75	1.44	12 (33.3)	1.42	0.60	0	< 0.0001	0.01
**Talking with the Healthcare Team**								
Q10 I understand my doctor’s/pharmacist’s instructions about the medicines I take.	1.81	0.91	5 (13.9)	1.61	0.89	3 (8.3)	0.37	1.0
Q11 My doctor/pharmacists and I work together to make decisions.	3.56	1.48	23 (63.9)	1.50	0.69	2 (5.56)	< 0.0001	< 0.0001
Q12 I am able to read and understand pill bottle labels.	2.19	1.24	13 (36.4)	1.42	0.83	3 (8.3)	0.003	0.13
**Taking Medicines**								
Q13 Taking medicines more than once a day is inconvenient.	2.56	1.44	11 (30.6)	1.39	0.76	1 (2.8)	< 0.0001	0.24
Q14 I have to take too many medicines a day.	2.33	1.35	8 (22.2)	1.39	0.76	2 (5.6)	< 0.001	0.30
Q15 It is hard for me swallow the pills I have to take.	1.56	0.68	0	1.08	0.36	0	< 0.001	1.0
**Have you …**								
Q16 Taken a medicine more or less often than prescribed?	1.78	1.36	6 (16.7)	1.28	0.69	3 (8.3)	0.06	1.0
Q17 Skipped or stopped taking a medicine because you didn’t think it was working?	1.42	1.01	4 (11.1)	1.03	0.16	0	0.03	0.29
Q18 Skipped or stopped taking a medicine because it made you feel bad?	1.28	0.84	2 (5.6)	1.06	0.16	1 (2.8)	0.15	1.0
Q19 Skipped, stopped, not refilled, or taken less medicine because of the cost?	1.03	0.16	0	1.0	0	0	0.32	1.0
Q20 Not had medicine with you when it was time to take it?	1.92	1.30	5 (13.9)	1.11	0.31	0	< 0.001	0.15

The study period was 26 ± 2 weeks. ^*^Paired t-test for the change in mean score from baseline to end. ^‡^Fisher’s exact test for the prevalence of barriers between baseline and end

## Discussion

This study was the first to investigate whether pharmacist involvement in the inhaler choice was beneficial for improving lung function in patients with COPD. The improvement in FEV_1_ in this study was > 0.35 L. This improvement is ≥1.5 times more than the expected maximum improvement in FEV_1_ with the inhaler therapy (approximately 0.2 L [20, 21)]). Pharmacist workload in inhaler choice (approximately 30 min) could be acceptable because a previous study [9)] reported that 15–30 min was spent in patient education and shared decision-making.

In this study, the pharmacists focused on the improvement of medication continuity. Although predictability of poor adherence of inhaler therapy using ASK-20 may be inferior among patients with COPD than patients with asthma [22)], the results for ASK-20, especially the results for Question 7 (‘I feel confident that each of my medicines will help me’), Question 8 (‘I know if I am reaching my health goals’) and Question 11 (‘My doctor/pharmacists and I work together to make decisions’), suggest that the significant improvement in lung function may have been associated with shared decision-making. The patients were able to choose the inhaler for themselves from inhalers selected by the pharmacists referring to the inhaler choice protocol. This patient participation in the inhaler choice may result in consistent improvement of FEV_1_ in all COPD stages.

A recent study reported that CAT responders, defined as those with a decrease of ≥2 points from baseline in the CAT [23)], had a significantly lower rate of acute exacerbation than non-responders. The prevalence of CAT responders in this study was 86%, which was considerably higher than that reported in previous interventional studies, of 36.7–55% [23–26)].

Essentially, no patient experienced acute exacerbation. Previous studies have reported that a mMRC dyspnea score ≥2 points was a significant predictor of COPD exacerbation [27, 28)]. In this study, the percentage of patients with mMRC dyspnea scores ≥2 points decreased significantly from 72% at baseline to 17% at the end of the study period. These results suggest that pharmacist involvement in the inhaler choice may contribute to a reduction in the risk of exacerbation.

We acknowledge some limitations of this study. First point is most important limitation. Regarding the improvement of patient outcome, although the pharmacists were given discretion to choose an inhaler under the inhaler choice protocol (Fig. 2, Process 5), we cannot clearly distinguish the impact between pharmacist involvement in the inhaler choice and originally developed inhaler choice protocol. Moreover, the generalizability of the results may be a substantial limitation of this study because they were based only on the works of three pharmacists from a single institution. That is, we cannot investigate the pharmacist competency framework for involvement in the choice of inhaler. Second, to improve COPD patient outcomes, multidisciplinary treatment strategies, such as pulmonary rehabilitation and nutritional counselling, are essential [29)]. Although none of the patients in the present study received intervention from physiotherapists and nutritionist, unmeasured factors related to respiratory rehabilitation and nutritional counselling may be potential confounders. Finally, although we conducted the single arm, non-comparison trial because of serious discrepancies in comparison with the historical control, such as available inhaler, non-pharmacological therapies, and recommended-inhaled medication each GOLD stages, a comparison study between the PBPM and routine practice should be conduct to investigate the impact of pharmacist involvement in the inhaler choice. To return the exploratory results in this study to routine pharmaceutical care, a multi-institutional comparison study is warranted to confirm the feasibility of pharmacist involvement in the choice of inhaler.

## Conclusion

In conclusion, pharmacist involvement in the inhaler choice for patients with newly diagnosed COPD was associated with improved lung function, health status, clinical symptoms, and adherence to inhaler therapy. In the management of COPD, pharmacist may play an important role in inhaler choice, with patient-centered care, when planning inhaled medication. Shifting task of choosing inhaler from physician to hospital pharmacist may be performed effectively and safely with an inhaler choice protocol.

## Supplementary Information

**Additional file 1 Fig. S1.** Patient flow diagram. **Table S1.** The pharmacist’s choice of inhaler for each patient and the factors contributing to the choice. **Table S2.** Choice of inhaler at baseline. **Table S3.** Sub analysis of the changes in FEV_1_ from baseline to the end of the study period for each COPD stage.

## Data Availability

The datasets used and/or analysed during the current study are available from the corresponding author on reasonable request.
