# Supplementary material for: Pharmacist involvement in the inhaler choice improves lung function in patients with COPD: a prospective single-arm study
Source: J Pharm Health Care Sci. 2021 Aug 2;7:28. doi: 10.1186/s40780-021-00211-0 (PMC8327440; doi:10.1186/s40780-021-00211-0)
Supplement: Supplementary file 1 — Additional file 1 Fig. S1. Patient flow diagram. Table S1. The pharmacist’s choice of inhaler for each patient and the factors contributing to the choice. Table S2. Choice of inhaler at baseline. Table S3. Sub analysis of the changes in FEV1 from baseline to the end of the study period for each COPD stage. [file 40780_2021_211_MOESM1_ESM.docx]

**Pharmacist involvement in the inhaler choice improves lung function in patients with COPD: A prospective single-arm study.**

**Authors’ full names:** Eiji Shiwaku^1^, Satoshi Dote^1*^, Shinobu Kaneko^1^, Chisato Hei^1^, Masaki Aikawa^2^, Yuki Sakai^2^, Takahiro Kawai^2^, Shigeaki Iwatsubo^2^, Michinobu Hashimoto^2^, Teppei Tsuneishi^2^, Tomoko Nishimura^2^, Toshiyuki Iwata^2^, Daiki Hira^3,4^, Tomohiro Terada^3^, Takashi Nishimura^2^, Yuka Kobayashi^1^

**Authors’ affiliations:**

^1^Department of Pharmacy, Kyoto-Katsura Hospital, Kyoto, Japan

^2^Department of Respiratory Medicine, Kyoto-Katsura Hospital, Kyoto, Japan

^3^Department of Pharmacy, Shiga University of Medical Science Hospital, Shiga, Japan

^4^College of Pharmaceutical Sciences, Ritsumeikan University, Shiga, Japan

**Corresponding author:**

Satoshi Dote

Department of Pharmacy, Kyoto-Katsura Hospital. 17, Yamadahiraocho, Kyoto-shi Nishikyo-ku, Kyoto, 615-8256, Kyoto, Japan

Email: [everyday.is.a.new.day.1981@gmail.com](mailto:everyday.is.a.new.day.1981@gmail.com)

**Figure S1** Patient flow diagram

Discontinued (n = 12, 20%)

- Lost to follow-up (n = 6, 10%)
- Died (n = 4, 6.7%)
- Pneumonectomy (n = 2, 3.3%)

Completed follow-up at study end

(n = 48, 80.0%)

Newly initiated inhaler therapy for COPD

from 1 April 2016 to 31 March 2018

(n = 60)

Analyzed

(n = 36, 75.0%)

Met the additional exclusion criteria (n = 12, 25%)

- Inpatient (n = 2, 4.2%)
- Lung Cancer (n = 8, 16.7%)
- Interstitial pneumonia (n = 2, 4.2%)

Abbreviations: COPD, chronic obstructive pulmonary disease

**Table S1**. The pharmacist’s choice of inhaler for each patient and the factors contributing to the choice

| Case | Age (y)/ Sex | COPD stage | Indicated inhaled medication | Inspiratory flow rate (L/min) | Can maintain sitting position | Difficulty using fingers^*^ | Muscle weakness^*^ | Poor inhalation synchronization^*^ | Difficulty sticking mouth over inlet | Poor eyesight^*^ | Difficulty with hearing^*^ | Availability of caregiver | Patient’s concerns/ preferences | Pharmacist’s concerns | Final choice |
| --- | --- | --- | --- | --- | --- | --- | --- | --- | --- | --- | --- | --- | --- | --- | --- |
| 1 | 46/M | II | LAMA | >30 | Yes | No | No | No | No | No | No | Not required | High portability and kit device | None | Respimat^®^ |
| 2 | 76/M | III | LAMA | >30 | Yes | No | No | No | No | No | No | Not required | Inhalation twice a day is not possible | Preference for kit device because patient hunted in the mountains for months | Ellipta^®^ |
| 3 | 66/M | II | LAMA | >30 | Yes | No | No | No | No | No | No | Not required | Visual and audible confirmation | None | Breezhaler^®^ |
| 4 | 81/M | III | LABA/ LAMA | >30 | Yes | Yes | No | No | No | No | No | No | None | Difficult to open capsule sheet | Ellipta^®^ |
| 5 | 74/M | II | LAMA | >30 | Yes | No | No | No | No | No | No | Not required | Preference for kit device | Prioritized patient’s preference within kit devices | Respimat^®^ |
| 6 | 76/M | III | LABA/ LAMA | >30 | Yes | No | No | No | No | No | No | Not required | Visual confirmation | None | Breezhaler^®^ |
| 7 | 84/M | II | LABA/ LAMA | >30 | Yes | Yes | Yes | No | No | No | Yes | No | None | Preference for simple handling | Ellipta^®^ |
| 8 | 90/M | II | LABA | >30 | Yes | No | No | No | No | No | Yes | Yes | None | Visual confirmation for caregiver | Breezhaler^®^ |
| 9 | 76/M | III | LABA/ LAMA | >30 | Yes | Yes | Yes | No | No | Yes | No | Yes | None | Because of hemiplegia, easy to handle | Ellipta^®^ |
| 10 | 65/M | III | LABA/ LAMA | >30 | Yes | Yes | No | No | No | Yes | No | Not required | None | Preference for simple handling | Ellipta^®^ |
| 11 | 77/F | II | LABA/ LAMA | >30 | Yes | Yes | No | No | No | No | No | Not required | None | Considering the future addition of ICS | Ellipta^®^ |
| 12 | 85/M | III | LABA/ LAMA | >30 | Yes | No | No | No | No | Yes | No | No | None | Patient could not open capsule sheet | Ellipta^®^ |
| 13 | 72/F | II | LABA/ LAMA | >30 | Yes | No | No | No | No | No | No | Not required | None | Prioritized patient’s preference | Ellipta^®^ |
| 14 | 80/M | II | LABA/ LAMA | >30 | Yes | No | No | No | No | No | No | Not required | As easy to handle as possible | None | Ellipta^®^ |
| 15 | 76/M | I | LABA/ LAMA | >30 | Yes | No | No | No | No | No | No | Not required | Visual confirmation and as cheap as possible | None | Breezhaler^®^ |
| 16 | 72/M | III | LABA/ LAMA | >30 | Yes | Yes | No | No | No | No | No | No | None | Patient unable to set inhaler capsule to device | Ellipta^®^ |
| 17 | 64/M | II | LABA | >30 | Yes | No | No | No | No | No | No | Not required | Preference for once daily | None | Breezhaler^®^ |
| 18 | 77/F | II | LAMA | <20 | Yes | No | No | Yes | Yes | No | Yes | Yes | None | The patient felt uncomfortable when sitting | Respimat^®^ |
| 19 | 72/M | III | LABA/ LAMA | >30 | Yes | No | No | No | No | No | No | Not required | Preference for once daily | Prioritized patient’s preference | Ellipta^®^ |
| 20 | 85/M | I | LABA | >30 | Yes | Yes | No | No | No | No | Yes | Not required | Visual confirmation | Considering future changes to LAMA or LABA | Breezhaler^®^ |
| 21 | 77/M | III | LABA/ LAMA | >30 | Yes | No | No | No | No | No | No | Not required | Preference for kit device | None | Ellipta^®^ |
| 22 | 72/F | II | LABA/ LAMA | >30 | Yes | No | No | No | No | No | No | Not required | Visual confirmation | None | Breezhaler^®^ |
| 23 | 66/M | II | LABA/ LAMA | >30 | Yes | No | No | No | No | No | No | Not required | Insecure about inhalation and felt mist medication may be unreliable | None | Ellipta^®^ |
| 24 | 58/M | II | LAMA | >30 | Yes | No | No | No | No | No | No | Not required | Preference for compact size and easy to handle | Preference for once daily inhalation, because of lifestyle | Ellipta^®^ |
| 25 | 70/M | II | LABA/　LAMA | >30 | Yes | No | No | No | No | Yes | No | Not required | Insecure about inhalation and preference for once daily | Insecure about daily inhalation and short inspiratory time | Ellipta^®^ |
| 26 | 72/M | II | LAMA | >30 | Yes | No | No | No | No | No | No | Not required | Preference for once daily | None | Breezhaler^®^ |
| 27 | 63/M | III | LABA/ LAMA | >30 | Yes | No | No | No | No | No | No | Not required | Preference for simple device handling | None | Ellipta^®^ |
| 28 | 71/M | II | LABA/ LAMA | >30 | Yes | No | No | No | No | No | No | Not required | Visual confirmation and preference for kit device | Unable to inhale DPI (by using trainer) | Respimat^®^ |
| 29 | 76/M | II | LABA/ LAMA | >30 | Yes | No | No | No | No | No | No | Not required | High portability | Preference for once daily | Ellipta^®^ |
| 30 | 55/M | I | LAMA | >30 | Yes | No | No | No | No | No | No | Not required | Preference for once daily | Prioritized patient’s preference | Ellipta^®^ |
| 31 | 69/ F | II | LABA/ LAMA | <20 | Yes | No | No | No | No | No | No | Not required | High portability | Low inspiratory flow rate | Respimat^®^ |
| 32 | 53/M | II | LAMA | >30 | Yes | Yes | No | No | No | No | No | Yes | Insecure about inhalation | Unsuited to handling Respimat twice daily | Ellipta^®^ |
| 33 | 70/M | II | LABA | >30 | Yes | No | No | No | No | No | No | Not required | Preference for once daily | None | Breezhaler^®^ |
| 34 | 69/M | IV | LABA/ LAMA | >30 | Yes | Yes | No | No | No | Yes | Yes | Yes | None | Visual confirmation, and unstable and short inspiratory flow rate | Breezhaler^®^ |
| 35 | 77/M | II | LAMA/ LABA | >30 | Yes | No | No | No | No | No | No | Not required | Preference for once daily | Felt uncomfortable with Ellipta inhalation | Respimat^®^ |
| 36 | 69/M | I | LAMA | >30 | Yes | No | No | No | No | No | No | Not required | Visual confirmation, easy handling, and preference for once daily | None | Ellipta^®^ |

^*^The pharmacists assessed patient's physical function subjectively.

Abbreviations: LAMA, long-acting muscarinic antagonist; LABA, long-acting beta 2-agonist; ICS, inhaled corticosteroid.

**Table S2**. Choice of inhaler at baseline

| Drug class and device | | n = 36 |
| --- | --- | --- |
| LAMA | Breezhaler^®^ | 2 |
|  | Ellipta^®^ | 5 |
|  | Respimat^®^ | 3 |
|  | Total | 10 |
| LABA | Breezhaler^®^ | 4 |
|  | Total | 4 |
| LABA/LAMA | Breezhaler^®^ | 4 |
|  | Ellipta^®^ | 15 |
|  | Respimat^®^ | 3 |
|  | Total | 22 |

Abbreviations: LAMA, long-acting muscarinic antagonist; LABA, long-acting beta 2-agonist; ICS, inhaled corticosteroid

| COPD stage | n | Baseline (L) | Change in FEV_1_ from baseline to end (L) | 95% Confidence Interval | P value^*^ |
| --- | --- | --- | --- | --- | --- |
| Stage I | 4 | 2.41 ± 0.41 | 0.32 ± 0.10 | 0.14 to 0.51 | 0.011 |
| Stage II | 21 | 1.70 ± 0.44 | 0.31 ± 0.21 | 0.21 to 0.40 | <0.0001 |
| Stage III | 10 | 1.14 ± 0.19 | 0.57 ± 0.40 | 0.27 to 0.87 | 0.002 |
| Stage IV | 1 | 0.90 | 0.48 | - | - |

**Table S3**. Sub analysis of the changes in FEV_1_ from baseline to the end of the study period for each COPD stage

The study period was 26 ± 2 weeks. Data are presented as mean ± SD.

^*^Paired t-tests for the change in mean FEV_1_ from baseline to end.
